# Squid Ink Polysaccharides Protect Human Fibroblast Against Oxidative Stress by Regulating NADPH Oxidase and Connexin43

**DOI:** 10.3389/fphar.2019.01574

**Published:** 2020-01-17

**Authors:** Ying Chen, Huazhong Liu, Hao Huang, Yuetang Ma, Ruihua Wang, Yong Hu, Xiufen Zheng, Chunmei Chen, Hongfeng Tang

**Affiliations:** ^1^ Department of Dermatology, Nanchong Central Hospital, the Second Clinical Medical College of North Sichuan Medical College, Nanchong, China; ^2^ Department of Dermatology, Shunde Hospital, Southern Medical University (The First People’s Hospital of Shunde Foshan), Foshan, China; ^3^ College of Chemistry and Environment, Guangdong Ocean University, Zhanjiang, China

**Keywords:** squid ink polysaccharides, fibroblasts, oxidative stress, NADPH oxidase, connexin43

## Abstract

Oxidation injury to skin is one of the main reasons for skin aging. The aim of the present study was to explore the protective effect of squid ink polysaccharides and its mechanism of action against H_2_O_2_-induced dermal fibroblast damage. Our results show that squid ink polysaccharides effectively reduce the fibroblast oxidative damage mediated by the up-regulation of NADPH oxidase and Connexin43. Concurrently, squid ink polysaccharides decrease the ROS induced up-regulation of MMP1 and MMP9 to decrease MMP-mediated skin aging. Therefore, we hypothesize that squid ink polysaccharides play an antioxidant role by inhibiting the expression of NADPH oxidase and connexin43. This provides a new target for the effective clinical prevention and treatment of oxidative skin damage.

## Introduction

Skin aging is the most common human aging. Skin aging not only seriously affects beauty but is also associated with many skin diseases (Bosset et al., 2002). With the development of the social economy, approaches to delay the onset of skin aging have gained attention worldwide; therefore, preventing and delaying skin aging have become popular modern medical research topics. Oxidation injury of the skin is one of the main reasons for skin aging (Noh et al., 2016). To prevent skin oxidative damage and protect the normal structure and function of skin tissue, it is necessary to postpone skin aging.

Reactive oxygen species (ROS) are oxygen-containing small species, including ozone (O_3_), hydroxy radical (OH^•^), singlet oxygen (^1^O_2_), hydrogen peroxide (H_2_O_2_), and superoxide anion radical (O_2_^•−^) (Sosa et al., 2013). Research has shown that H_2_O_2_ is the main ROS and can directly or indirectly damage cells and induce apoptosis and necrosis (Bedard and Krause, 2007; Morry et al., 2016); therefore, it was used to induce oxidative stress *in vitro* models. ROS also play an important role in skin aging caused by UV light (Fisher et al., 2002). Previous studies showed that when intracellular redox disrupted the balance between the clearance and production of ROS, excessive ROS accumulation resulted in oxidative stress and induced oxidative damage (Sohal and Allen, 1990; Sarkar and Fisher, 2006). In particular, ROS induced the increased expression of matrix metalloproteinases (MMPs) and promoted the degradation of collagen and elastin in the extracellular matrix (ECM), thereby causing skin to lose elasticity and become tough, eventually leading to the occurrence of skin aging (Sbardella et al., 2012; Lee et al., 2013). Many studies have shown that the NADPH oxidase (Nox) family is crucial for the induction of ROS (Babior, 1999) and that the Nox source of ROS was involved in the development of a variety of diseases (Gill and Wilcox, 2006; Miller et al., 2006; Bedard and Krause, 2007; Huang et al., 2017), but research on its role skin aging is lacking. Some scholars found that Connexin43 (Cx43) and Nox jointly influence the oxidative stress damage of kidney cells, and they suggested that Cx43 can be used as a new indicator of podocyte oxidative stress and as a novel therapeutic target to reduce podocyte damage (Kleniewska et al., 2012; Yan et al., 2012). However, the role of Cx43 in skin oxidative damage has not yet been reported. This study discusses the role of Nox and Cx43 in the oxidative damage of skin.

Active antioxidant ingredients in skin care products can partially protect the skin against oxidative damage. Many studies showed that adding antioxidants to cosmetics or using products for skin disease treatment that contain antioxidants can effectively prevent ultraviolet-light-mediated skin damage (D'Angelo et al., 2012; Chang et al., 2014; Hu et al., 2016; Nakajima et al., 2016). In the past, the selection of natural antioxidants was mostly from plants. Anthocyanins, resveratrol, and green tea polyphenols extracted from plants have been widely studied and applied in many fields (Lim et al., 2011; Szczepanek et al., 2012). With the development of marine resources, abundant resources of antioxidants have been identified. Squid ink polysaccharides (SIP) is a type of highly functional active ingredient that is efficient and non-toxic and is extracted from sepia in the ocean. Its main chemical composition is melanin and protein polysaccharide complexes (Sasaki et al., 1997). Takaya et al. (1996) obtained three polysaccharide compositions, Illexins A, B, and C from SIP, including glucuronic acid (GlcA), fucose (Fuc), and N-acetyl galactose (GalNAc). Its chemical formula is [-3G1cAβ1-4(GalNAcα 1-3)Fucα1-]n. Recently, SIP was shown to have antioxidative, antitumor, antibacterial, and chemotherapy protection effects, and the antioxidant function garnered much attention (Guo et al., 2014; Zuo et al., 2015). Studies have shown that SIP *in vitro* effectively removes DPPH and HO• ROS and can effectively relieve cyclophosphamide (CP)-induced oxidative damage of multiple organs and tissues, such as bone marrow, heart, liver, and kidney (Shanholtz, 2001; Zhong et al., 2009). It also inhibits the increase of lipid peroxide malondialdehyde (MDA) induced by CP and restores the activity of the catalase (CAT) and antioxidant enzymes superoxide dismutase (SOD) (Le et al., 2015). However, there is no research on the effect that SIP has on skin oxidative damage. Therefore, our study is the first to explore the antioxidative effect of SIP *in vitro* using human dermal fibroblasts (HDFs). In the present study, we investigated the ability of SIP to protect HDFs from H_2_O_2_-induced oxidative stress and apoptosis.

## Materials and Methods

### Reagents

Purchase live squid from the aquatic product market and kill the squid to obtain fresh ink sacs, then store the sac at -28°C for future use. The ink collected from the ink bag was thawed at 4°C, resuspended in PBS(pH 6.7), then ground and sonicated. The resulting ink solution was stored at 4°C for 24 h and then centrifuged (14,000 g) at 4°C for 1 h. The supernatant was subjected to enzymolysis with 1% papain in PBS (pH 6.7) at 60°C for 24 h and was then mixed with a 1/4 volume liquid mixture of chloroform and n-butanol (v/v, 4/1), followed by stirring for 30 min on a magnetic stir plate. After centrifugation (5,000 g) for 15 min, the supernatant was again digested with papain and the digestion process was repeated twice. The resulting supernatant was precipitated with four volumes of absolute ethanol and lyophilized under vacuum. SIP preparation was as follows: 100 mg SIP was dissolved in 1 ml of PBS, 100 mg/ml mother liquor was prepared, and samples were aliquoted in 1.5 ml Ep tubes and stored at -20°C (Le et al., 2015). According to the experimental needs for different concentrations, the SIP was diluted with PBS to the corresponding concentration in advance. MTT solution and 2',7'-dichlorofluorescein diacetate diacetate (DCFH-DA) were purchased from Sigma-Aldrich Corporation (USA). For the Annexin V-FITC apoptosis detection assay, we used a kit purchased from Biovision (Mountain View, CA). ROS, MDA, SOD, and GSH-PX assay kits were obtained from Nanjing Jiancheng Bioengineering Institute (China). The primary antibodies anti-Nox1, anti-Nox2, anti-Nox4, anti-Nox5, and anti-Connexin43 were purchased from Abcam Corporation (USA), and anti-MMP1 and anti-MMP9 were purchased from Proteintech Corporation (China). The secondary antibodies, anti-rabbit-HRP and anti-goat-HRP, were purchased from Bioword Corporation (China).

### Cell Culture

HDFs isolated from the foreskins of four men (age 16–25) following circumcision surgery were routinely cultured in DMEM supplemented with 10% newborn calf serum (Gibco; USA) with 100 U/ml penicillin and 100 mg/ml streptomycin, following homogenization of the tissue. Cells were maintained at 37°C with 5% CO_2_ in a humidified incubator. When the cells reached 80% confluence, the cells were harvested with trypsin and seeded in a 6-well plate at a density of 1×10^5^ cells/well at 37°C. After 48 h of incubation, the cultured medium was removed. The cultured cells were stored in a nitrogen tank. Cells between passages 4 and 10 were used in subsequent experiments. The Ethics Committee of Southern Medical University affiliated with the First People's Hospital of Shunde approved all of the procedures in this study.

### Cell Treatments and Measurement of Cell Viability

Cells were cultured in 96-well plates and maintained in medium for 48 h. Cells at 60–80% confluence were washed with PBS before treatment with H_2_O_2_ at different concentrations (200, 400, 600, 700, and 800 μmol/L). Then, cells were incubated for 24 h for viability assays. The appropriate concentration of H_2_O_2_ was selected and then SIP was added at different concentrations (0.125, 0.25, 0.5, and 1 mg/ml) to prevent oxidative damage.

Cell viability was detected by MTT assay (Carmichael et al., 1987). Briefly, 20 μl/well of MTT reagent (5 mg/ml) was added to the cells after treatment and incubated for 4 h. Then, the medium was aspirated and replaced with 150 μl/well of DMSO. Measurements were collected using a Multiscan Spectrum at 490 nm. Cell viability is proportional to the absorbance measured.

### Detection of Intracellular ROS

The DCFH-DA fluorescent dye was used for ROS analysis (Rosenkranz et al., 1992). Following treatment with H_2_O_2_ with or without SIP, the cells were collected and incubated with 10μmol/L DCFH-DA at 37°C for 30 min. Then the cells were washed in PBS three times and the fluorescent signal intensity was analyzed using a flow cytometer (LSR Foressa, Becton, USA) at excitation and emission wavelengths of 485 and 525 nm.

### Apoptosis Detection Assay

Apoptosis was determined using an Annexin V-fluorescein isothiocyanate (FITC)/propidium iodide (PI) staining procedure. After treatment with H_2_O_2_ and incubation with SIP, cells were harvested and washed twice with ice-cold PBS and then incubated with annexin V-FITC and PI. The fluorescent signal intensity was analyzed using a flow cytometer with excitation and emission wavelengths of 488 and 530 nm.

### Measurement of MDA Content and Activities of SOD and GSH-Px

The lipid peroxide malondialdehyde (MDA) content and enzyme activities of glutathione peroxidase (GSH-Px) and SOD were determined using specific assay kits purchased from Nanjing Jiancheng Bioengineering Institute and were performed according to the manufacturer's instructions. The samples were detected by a Multiscan Spectrum (SpectraMax M5, CA, USA) for SOD activity at 450 nm, GSH-PX activity at 532 nm, and MDA content at 405 nm, respectively.

### Western Blot Analysis

At a density of 1×10^6^ cells/ml, the cells were treated with nothing, 700 μmol/L H_2_O_2_ alone, or a combination of 700 μmol/L H_2_O_2_ and 0.5 mg/ml SIP at 37°C for 24 h and were then harvested at the indicated time points. After the lysis procedure, the lysate was centrifuged at 12,000 g for 15 min at 4°C. The protein concentration of the supernatant was quantified using a Bicinchoninic Acid Protein Assay Reagent (Beyotime Institute of Biotechnology). Protein (40–50μg) from each sample was separated by 30% SDS-PAGE and transferred to a polyvinylidene fluoride membrane. Each membrane was subsequently incubated with the primary antibody overnight at 4°C each antibody at a dilution of 1:1,000, then washed 3 times for 5 min each, and then incubated with the secondary antibody for 1–2 h at room temperature. GAPDH expression was used as the reference band. The protein expression rate was quantified using the ImageJ software.

### Statistical Analysis

The experiments were repeated at least three times, and the values are expressed as the means ± SD. The statistical analysis was conducted with SPSS 20.0. A paired-samples *t* test was used for the statistical analysis of differences between two groups. For multiple comparisons, data were tested using one-way ANOVA and, subsequently, with Tukey or Dunnet multiple comparison test. P values less than 0.05 were considered statistically significant.

## Results

### Protective Effect of SIP Against the H_2_O_2_-Induced Decrease of Cellular Activity in HDFs

**Figure 1:**
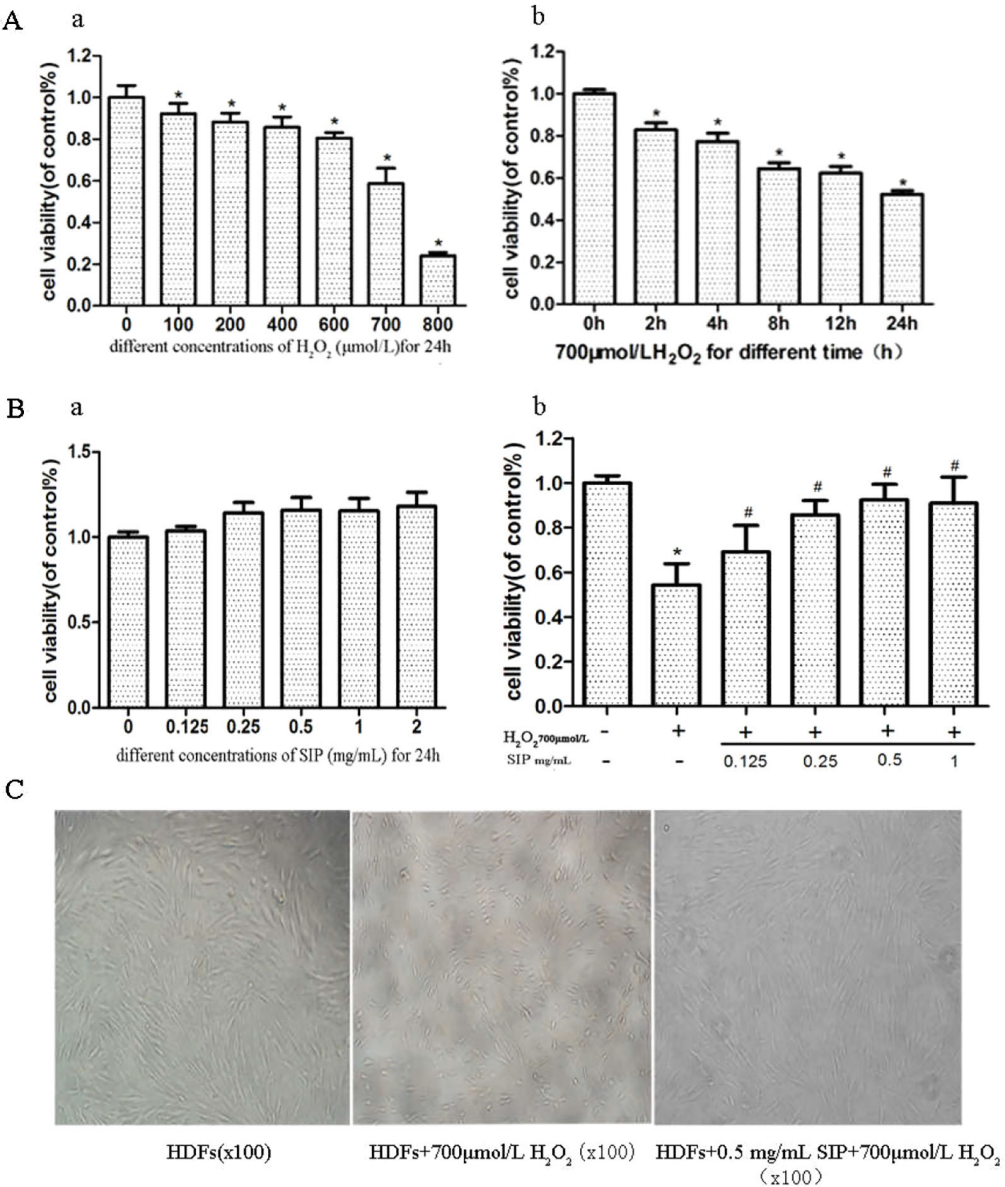
Protective effect of SIP against H_2_O_2_-induced cell activity decrease in HDFs. **(A)** The effect of H_2_O_2_ on the activity of HDFs. The results are presented as the mean ± standard error of the mean (%), n = 5; *p < 0.05. **(B)** Effect of different concentrations of SIP on the activity of HDFs with or without H_2_O_2_ treatment. Results are presented as the mean ± standard error of the mean (%), n = 5; *p < 0.05 vs NC, ^#^ *p* < 0.05 vs H_2_O_2_ alone. **(C)** Effect of SIP on the morphology of HDFs with H_2_O_2_ treatment (100×).

H_2_O_2_ inhibited the survival of HDFs in a dose- and time-dependent manner (**Figure 1A**). Compared with the normal group after 700 μmol/L H_2_O_2_ treatment for 24 h, the cell viability decreased 40.8% (p < 0.05). The cell damage was obvious, and the cell survival rate was stable and moderate; therefore, it was suitable for establishing the oxidative damage model of HDFs.

After adding different concentrations of SIP (0.125, 0.25, 0.5, 1, and 2 mg/ml) to treat HDFs for 24 h, the cell viability increased slightly compared with the normal group, but the differences were not significant (P > 0.05) (**Figure 1B** a). This result indicated that SIP does not exhibit cell toxicity in normal HDFs.

Different concentrations of SIP (0.125, 0.25, 0.5, and 1 mg/ml) were used to pretreat HDFs for 2 h. Then, 700 μmol/L H_2_O_2_ was added and reacted for 24 h. MTT was used to determine cell viability. The results showed that compared with the H_2_O_2_ injury group, the SIP protection group had increased the cell viability (^#^P < 0.05) in a dose-dependent manner. However, at 0.5 and 1 mg/ml, there was no significant difference (P > 0.05) (**Figure 1B** b). Therefore, we selected 0.5 mg/ml SIP for subsequent experiments.

Cell morphology was observed using an inverted microscope (**Figure 1C**). Compared to the normal control group (NC group), 700 μmol/L H_2_O_2_ changed the HDFs morphology significantly, including cell shrinkage, volume decrease, and cell gap increases. After pretreatment with 0.5 mg/ml SIP for 2 h, the cell morphology was restored, the number of cells increased and the cell volume increased, and there was no significant difference in morphology with the normal group.

### Scavenging Activity of SIP Against H_2_O_2_-Generated Intracellular ROS in HDFs

**Figure 2:**
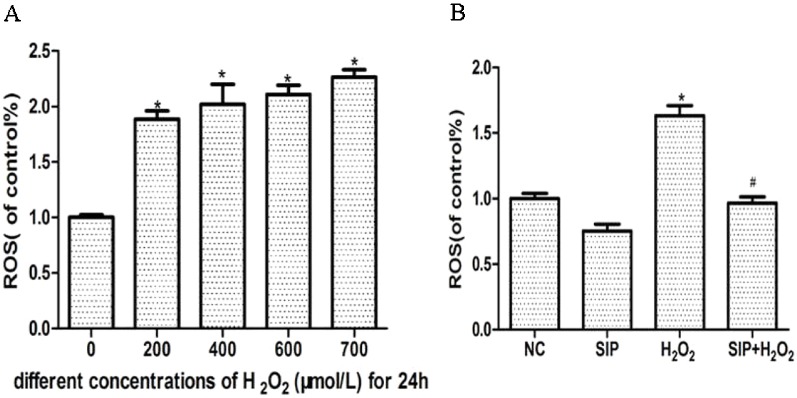
Scavenging activity of SIP against H_2_O_2_-generated intracellular ROS in HDFs. **(A)** H_2_O_2_ induces intracellular ROS. The results are presented as the mean ± standard error of the mean (%), n = 5; *p < 0.05. **(B)** SIP scavenges intracellular ROS. The results are presented as the mean ± standard error of the mean (%), n = 5; *p < 0.05 vs NC, ^#^ *p* < 0.05 vs H_2_O_2_ alone.

Different concentrations of H_2_O_2_ were used to treat HDFs for 24 h and increase the intracellular ROS levels in a concentration-dependent manner (**Figure 2A**). Pretreatment HDFs with SIP 0.5 mg/ml for 2 h then exposure to 700 μmol/L H_2_O_2_ for 24 h effectively reduced the H_2_O_2_-induced ROS (P < 0.05) (**Figure 2B**).

### Cytoprotective Effect of SIP Against H_2_O_2_-Induced Apoptosis

**Figure 3:**
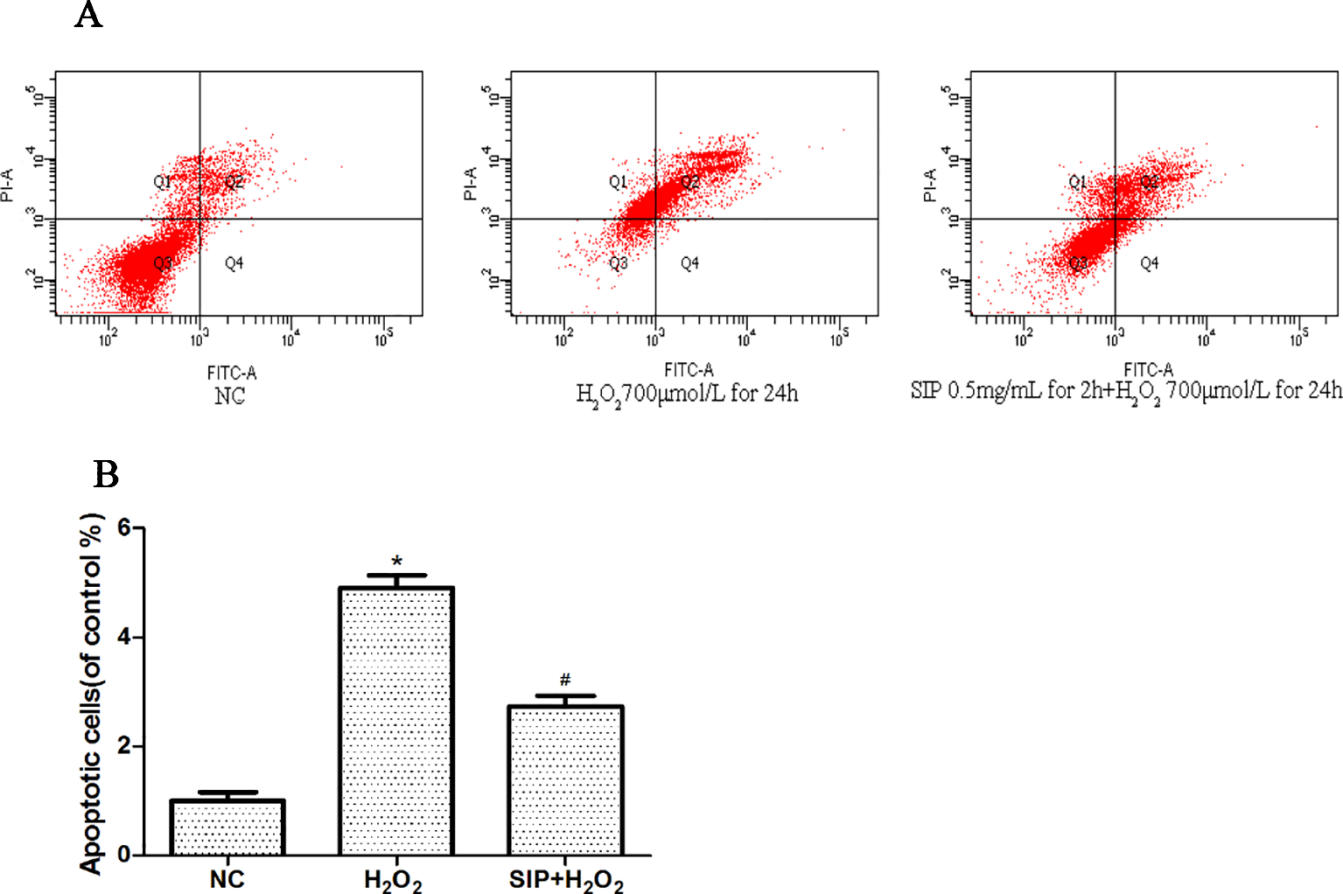
Cytoprotective effect of SIP against H_2_O_2_-induced apoptosis. **(A)** Flow cytometry results of apoptosis. **(B)** Apoptotic rate of HDFs. The results are presented as the mean ± standard error of the mean (%), n = 3; *p < 0.05 vs NC, ^#^ *p* < 0.05 vs H_2_O_2_ alone.

The effects on apoptosis were detected by flow cytometry and are shown in **Figures 3A, B**. As shown in **Figure3A**, when HDFs were exposed to 700 μmol/L H_2_O_2_ for 24 h, the apoptosis rate was 49.2%. However, in the pretreatment with SIP 0.5 mg/ml for 2 h, the apoptosis rate was only 31.4%. In **Figure 3B** after HDFs were exposed to 700 μmol/L H_2_O_2_ for 24 h, the apoptosis significantly increased (*P < 0.05), and pretreatment with SIP 0.5 mg/ml for 2 h significantly reduced the apoptosis caused by H_2_O_2_ (^#^P < 0.05).

### Effects of SIP on MDA Content and the Activity of SOD and GSH-Px in HDFs With H_2_O_2_ Damage

**Figure 4:**
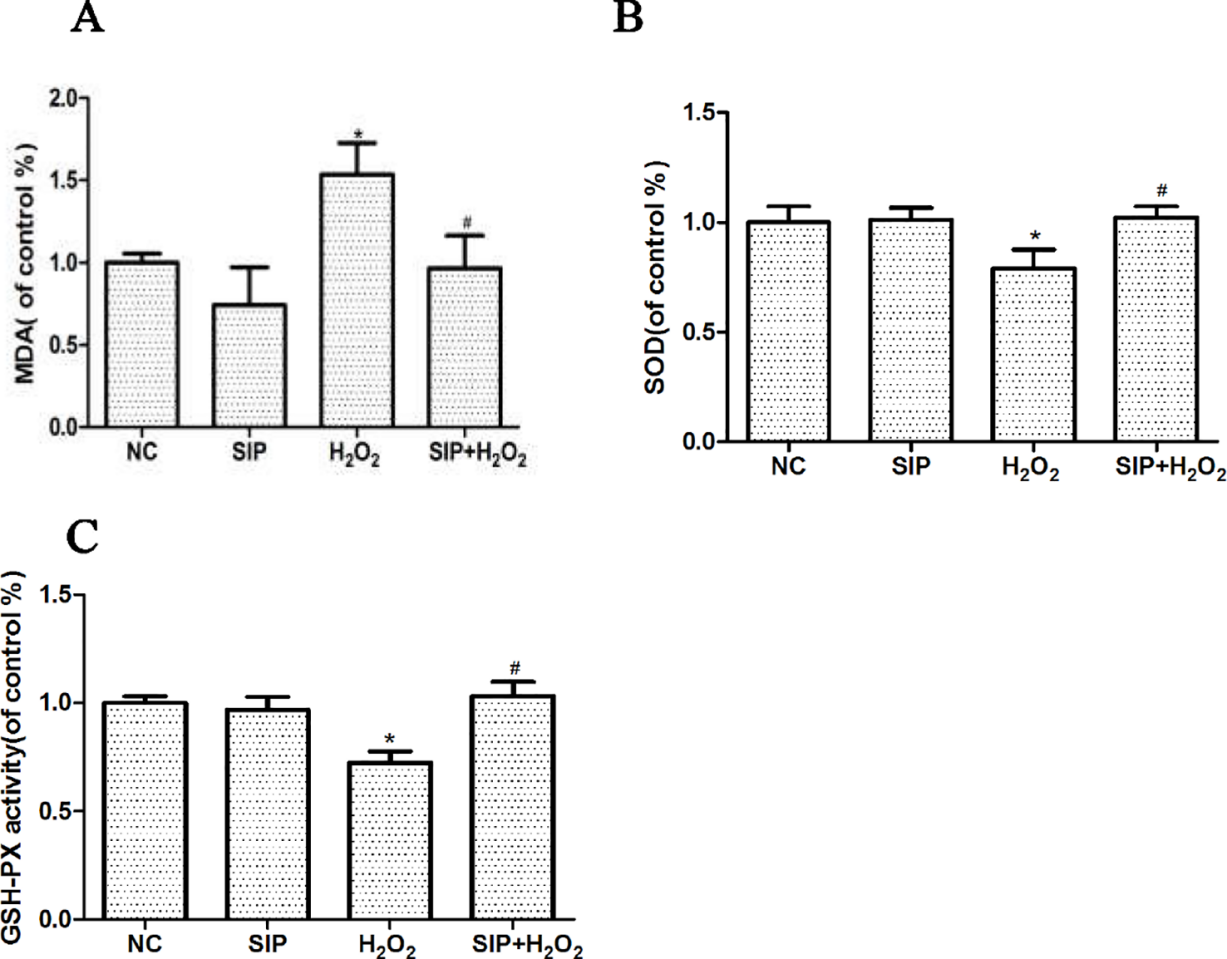
Effects of SIP on MDA content and the activity of SOD and GSH-Px in HDFs with H_2_O_2_ damage. **(A)** Effect of SIP on MDA content. **(B)** Effect of SIP on SOD activity. **(C)** Effect of SIP on GSH-Px activity. The results are presented as the mean ± standard error of the mean (%), n = 3; *p < 0.05 vs NC, ^#^ *p* < 0.05 vs H_2_O_2_ alone.

SIP 0.5mg/ml was used to pretreat HDFs for 2 h. Then 700 μmol/L H_2_O_2_ was added to each group for 24 h, according to the kit's instructions, and the MDA content, SOD, and GSH-Px activity changes were determined. After HDFs were exposed to 700μmol/L H_2_O_2_ for 24 h, the MDA content was increased, and the activity of SOD and GSH-Px was decreased. As shown in the **Figure 4**, pretreating HDFs with 0.5 mg/ml SIP effectively reduced the H_2_O_2_-induced increased in MDA, and increased the activity of SOD and GSH-Px (^#^P < 0.05).

### Effects of SIP on Related Protein Expression in HDFs

**Figure 5:**
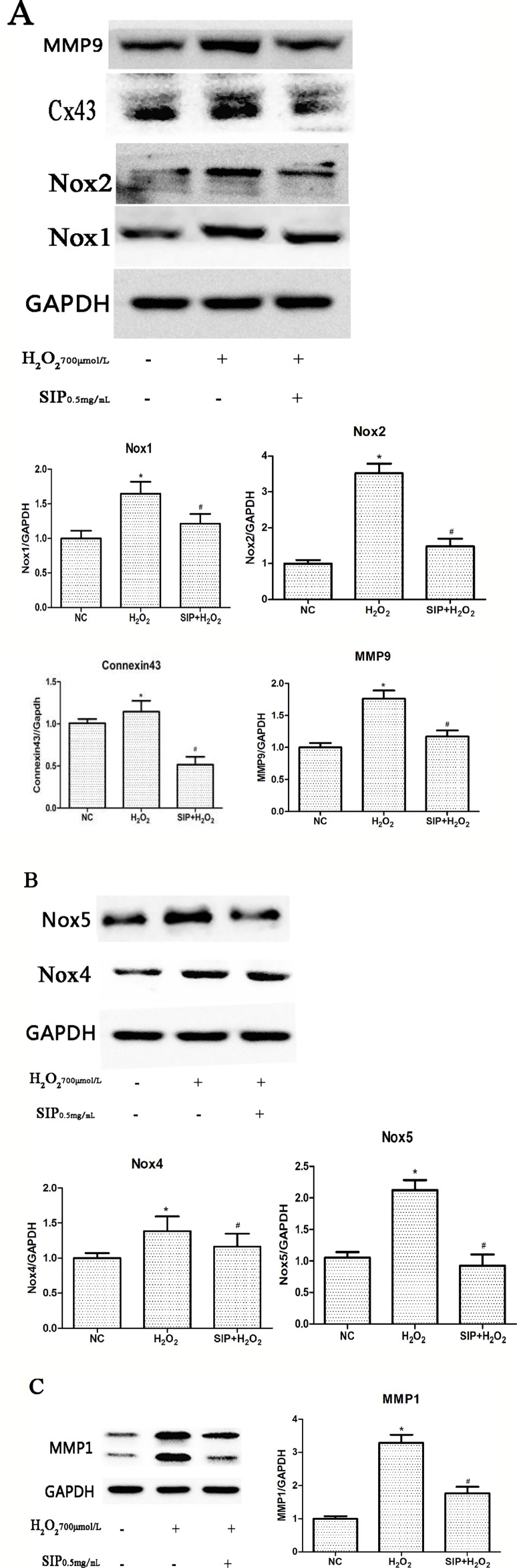
Effects of SIP on protein expression in HDFs. **(A)** Western blot results for NADPH oxidase(Nox1, Nox2), Connexin43 and MMP9. **(B)** NADPH oxidase(Nox4, Nox5) expression levels in HDFs. **(C)** Western blot results for MMP1. The results are presented as the mean ± standard error of the mean (%), n = 3; *p < 0.05 vs NC, ^#^ *p* < 0.05 vs H_2_O_2_ alone.

When HDFs were treated with 700 μmol/L H_2_O_2_ for 24 h, Western blot results showed that the expression of NADPH oxidase (Nox1, Nox2, Nox4, and Nox5), Connexin43, MMP1, and MMP9 were increased (*P < 0.05). Pretreating HDFs with SIP 0.5 mg/ml for 2 h reduced the H_2_O_2_-induced increasing of Nox1, Nox2, Nox4, Nox5, Connexin43, MMP1, and MMP9 expression (^#^P < 0.05) (**Figures 5A–C**). And we also found that when pretreatment HDFs with SIP 0.5 mg/ml for 2 h then exposure to 700 μmol/L H_2_O_2_ for 24 h, the expression of Cx43 was lower than the normal control group; we suspected the probable reason might be the SIP reduced the ROS produced in normal HDFs, which resulted in a drop in Cx43.

## Discussion

Skin aging can be divided into intrinsic or time aging, which is the aging process that affects all body organs, as well as external aging (photoaging) that occurs due to exposure to environmental factors (Noh et al., 2016). ROS, primarily arising from oxidative cell metabolism, play a major role in both chronological aging and photoaging of skin (Fisher et al., 2002). Most ROS are harmful to cells due to the accumulation of irreversible damages to polysaccharide, proteins, lipids and, most importantly, DNA, which leads to mutations and cell death, finally causing skin disease and aging (Leichert et al., 2008; Sun et al., 2012). Therefore, inhibition of ROS in cells is an effective method to prevent oxidative stress damage caused by cell metabolism and exogenous substances. H_2_O_2_ is an oxidant that is membrane permeable and is used to cause oxidative stress damage in a variety of cell culture models (Hou et al., 2015; Xia et al., 2015). In our experiment, we used exogenous H_2_O_2_ to induce HDFs oxidative stress damage. The results showed that H_2_O_2_ inhibited the survival of HDFs in a dose- and time-dependent manner (**Figure 1A**). Compared with the normal group, after treatment with 700μmol/L H_2_O_2_ for 24h the cell viability decreased 40.8% (p < 0.05), and the HDFs morphology changed significantly, including cell shrinkage, volume decrease, and cell gap increases (**Figure 1C**). Subsequently, H_2_O_2_ significantly increased the cell apoptosis rate (**Figures 3A, B**). Previous studies showed that ROS directly reflects the level of oxidative stress in cells (Ciarcia et al., 2010). Our experiment also indicated that the intracellular ROS level is consistent with cell damage, with a significant decrease in cell viability after the ROS level is increased. These results showed that with the increase of H_2_O_2_ first is the alteration of cell function (ROS increased), and when the damage accumulates, it will trigger irreversible cell damage (decreased cell viability and increased apoptosis).

The Nox family are proteins that transfer electrons across biological membranes (Bedard and Krause, 2007; Zhang et al., 2014). In general, NADPH oxidases generate superoxide at the plasma membrane and release it into the extracellular space, where it is converted into hydrogen peroxide. Valencia and Kochevar (2008) indicated that UVA activates Nox1-based NADPH oxidase to produce ROS that stimulate PGE2 synthesis and that Nox1 may be an appropriate target for agents designed to block UVA-induced skin injury. Spadoni et al. (2015) found that both Nox2 and Nox4 generate ROS in SSc fibroblasts and play a critical role in cell activation and DNA damage. Weyemi et al. (2015) provided evidence that the inactivation of Noxs protects cells from radiation-induced DNA damage and cell death. These studies suggest that NADPH oxidase and ROS-induced oxidative stress play key roles in skin injury and aging. Our results showed that the expression of NADPH oxidase (Nox1, Nox2, Nox4, and Nox5) was up-regulated after H_2_O_2_ stimulation (**Figures 5A, B**) and was consistent with the level of intracellular ROS and cell damage. These studies suggest that H_2_O_2_ may induce Nox activation, further leading to ROS accumulation and aggravating the oxidative stress response.

Lipid peroxidation malondialdehyde (MDA) is a byproduct of the oxidation of membrane lipids by ROS, which is an important biomarker for evaluating the level of oxidative stress in cells (Andriantsitohaina et al., 2012). SOD is a type of endogenous antioxidant enzymes that is induced by external stress. It can remove the ROS material within cells and plays an important role in maintaining cell oxidation and antioxidant balance (Weng et al., 2011). Glutathione peroxidase (GSH-Px) is another important enzyme for catalyzing H_2_O_2_ decomposition by catalyzing GSH with the H_2_O_2_ reduction reaction specifically, to protect the structure and function of the cell membrane (Ji et al., 2016). Therefore, SOD and GSH-Px can reflect the body's ability to resist oxidative damage. Our results showed that after H_2_O_2_ treatment, the MDA content was increased and the activity of SOD and GSH-Px was decreased (**Figures 4A–C**). This indicated that H_2_O_2_ caused severe oxidative stress, increased MDA production, and concurrently reduced HDFs self-defense and self-preservation functions, resulting in decreased SOD and GSH-Px activity and increased ROS content.

Connexins (Cxs) are a multigenic family of transmembrane proteins that form gap junctions, which allow small molecules and ions to flow from cell to cell (Zhang et al., 2015). Cx43 is one of the most important Cxs (Cina et al., 2009). Al Sovari et al. (2013) found that ROS activation resulted in mitochondrial injury, mitochondrial ROS production, a reduction in Cx43, and increased arrhythmic risk. Yang et al. (2014) showed that the Aldo-mediated up-regulation of Cx43 expression and the subsequent increases in ROS production and the Bax/Bcl-2 ratio are partially involved in podocyte injury; silencing Cx43 expression inhibited the Aldo-induced apoptosis of podocytes by reducing ROS production and down-regulating the Bax/Bcl-2 ratio. Previous studies showed that Cx43 plays an important role in maintaining the health of the body, but there are few studies of oxidative stress in skin. In this study, we investigated the role of Connexin43 in H_2_O_2_-induced oxidative damage in HDFs. Western blot results showed that with H_2_O_2_ treatment, Connexin43 expression increased in HDFs (**Figure 5A**). These results suggest that Connexin43 is involved in oxidative stress injury in fibroblasts.

MMPs, calcium- and zinc-dependent proteolytic enzymes, are responsible for extracellular protein degradation. They digest any physiological extracellular proteins (Wu et al., 2016). Therefore, MMPs are involved in several physiological and pathological processes. MMP activation leads to the excessive degradation of the skin structure, including elastin and collagen, causing a decrease of the dermal thickness and density, which causes poor skin elasticity, shrinkage, and other aging symptoms (Wall et al., 2003). Our results also showed that with H_2_O_2_ treatment, MMP1 and MMP9 expression increased in HDFs (**Figure 5A, C**). This suggests that oxidative stress injury up-regulates MMP expression.

Recently, many research groups evaluated herbal compounds and extracts for a protective effect against oxidative stress. Various phytochemicals, including tea polyphenols, catechins, glucosinolates, diallyl sulfides, and isothiocyanates, have been reported to induce oxidative stress (Lim et al., 2011; Szczepanek et al., 2012). In previous reports, it was shown that SIP, which was extracted from sepia in the ocean, has antioxidative (Guo et al., 2014), antitumor (Huang et al., 2012), antibacterial (Vate et al., 2015), and chemotherapy protection effects (Zhong et al., 2009), and its antioxidant function has garnered attention. However, there is no research on the effect of SIP in skin oxidative damage. Therefore, our experiment investigated the ability of SIP to protect HDFs from H_2_O_2_-induced oxidative stress and apoptosis.

First, we detected the toxicity of SIP to normal cells. Different concentrations of SIP (0.125, 0.25, 0.5, 1, and 2 mg/ml) were used to treat HDFs for 24 h, This result showed that SIP does not have cell toxicity in normal HDFs. In the H_2_O_2_-induced cell oxidative damage model, SIP pretreatment for 2 h increased the cell viability in a dose-dependent manner (**Figure 1B** b). Therefore, we hypothesized that SIP could effectively protect HDFs against H_2_O_2_-induced oxidative damage and preliminarily demonstrated the strong antioxidant activity of SIP. Then, we found that SIP reduces the level of ROS by H_2_O_2_ (**Figure 2B**). Therefore, we proposed that SIP reduces oxidative damage by reducing ROS production and aggregation. In addition, we found that SIP decreases the apoptosis of HDFs induced by H_2_O_2_ (**Figure 3**). At the same time, we measured the content of MDA and the activity of SOD and GSH-Px. The results showed that SIP reduces MDA and restores the activity of SOD and GSH-Px (**Figure 4**). We hypothesized that SIP up-regulates the levels of antioxidant enzymes in HDFs and improves their ability to eliminate free radicals, thereby reducing the level of lipid peroxidation and protecting against oxidative damage to HDFs. This study also examined the expression of MMP1 and MMP9. The results were consistent with our expectations. SIP effectively reduced the H_2_O_2_-induced up-regulation of MMP1 and MMP9 (**Figures 5A, C**). Then, SIP reduced the cell matrix decomposition and collagen and elastin reduction caused by the increase of MMP1 and MMP9 expression to protect the skin fibroblast activity and reduce the oxidative damage to the skin. Further studies indicated that pretreating HDFs with SIP significantly reduced the H_2_O_2_-induced increase in Nox1, Nox2, Nox4, Nox5, and Connexin43 expression (**Figures 5A, B**). We confirmed that SIP has strong antioxidant activity *in vitro* and that it resists oxidative stress induced by a variety of internal and external factors to protect the body from free radical damage. Furthermore, the mechanism of the antioxidant effect of SIP may be through the inhibition of NADPH oxidases and Connexin43 expression.

## Conclusion

In conclusion, this is the first study to show that squid ink polysaccharides (SIP) significantly relieves HDFs oxidative stress injury caused by H_2_O_2_. The fibroblast oxidative damage model that was established using H_2_O_2_ caused excessive ROS production in cells to induce HDFs oxidative damage, including increased MDA content and decreased SOD and GSH-PX activity, leading to the increased expression of NADPH oxidases (Nox1, Nox2, Nox4, Nox5), Connexin43, MMP1, and MMP9. SIP can effectively reduce fibroblast oxidative damage mediated by the up-regulation of NADPH oxidase and Connexin43. At the same time, SIP decreased the ROS induced up-regulation of MMP1 and MMP9 to ease MMP-mediated skin aging. Therefore, we propose that SIP overcomes the inhibition of the expression of NADPH oxidase and Connexin43 to exert its antioxidant effect. SIP has efficient antioxidant activity and may thus provide a new target for skin antioxidants and be useful as a therapeutic agent to mitigate oxidative stress.

## Data Availability Statement

All datasets generated for this study are included in the article.

## Ethics Statement

All of the tissue collection procedures of this study were approved by the Ethics Committee of Shunde Hospital, Southern Medical University (The First People’s Hospital of Shunde Foshan) in accordance with the principles of the Declaration of Helsinki. The written informed consent was obtained from all research participants.

## Author Contributions

HT was responsible for experimental design. YC, HL, HH, XZ, YH, RW, YM and CC were responsible for experimental operation. YM, YC, HH, and HT were responsible for writing the article.

## Funding

This research was supported by the Traditional Chinese Medicine Bureau of Guangdong Province (No. 20191317), Foshan Science and Technology Burea (FS0AA-KJ218-1301-0013), Clinical ResearchStart Program of Southern Medical University by High-level University Construction Funding of Guangdong Provincial Department of Education (No. PY2018N114), Scientific ResearchStart Plan of Shunde Hospital, Southern Medical University (No. SRSP2018014), Foshan Shunde Talent Development Service Center (Hongfeng Tang Expert Studio) and Foshan Medical Backbone Talents.

## Conflict of Interest

The authors declare that the research was conducted in the absence of any commercial or financial relationships that could be construed as a potential conflict of interest.
